# Book Review

**Published:** 1910-09

**Authors:** 


**Vetera et Nova.** By THOMAS LOGAN, M.D. Three Volumes. London: H. K. Lewis. 1910.

There is a certain pathos attaching to this collection of studies and essays, which the author had long been preparing for the press. His fatal illness left the actual publication in other hands, though no alterations or excisions have been made by the editors. So the three volumes are presented to us by Dr. Logan's friends as a memorial and a tribute, thereby perhaps avoiding any very searching criticism.

The essays represent the excogitations of a busy practitioner and public health officer with a taste for fine confused feeding in literature, and a love of travel which he was fortunately able to gratify freely; also he was a Scotsman from Burns' own Ayrshire, with the gift of expressive speech. His fertile brain and pen combined to furnish throughout a long life a running commentary on the progress of biological, physical and metaphysical science, but every essay and every page is deeply imbued with the personal metaphysic of the author.

The chronicling of scientific facts has not been supremely well effected; as a contribution to physical or metaphysical science it must be admitted to be valueless, but as a human document, as an exposition of the response of a single individual brain to a series of impressions garnered in from a vast field, it holds our attention.

These essays and studies may not, in fact, advance our knowledge or understanding of any of the subjects with which they deal, but they serve well to illustrate some results of unharnessed cerebration.

**Diseases of the Nervous System.** By H. Campbell Thomson, M.D., F.R.C.P. Pp. xv, 480. London: Cassell & Company, Limited. 1908.—This book is one in which the main phenomena of nervous diseases are gathered into a relatively small compass, the different diseases being clearly but shortly described. It is one which should be very useful to the student, for it is well illustrated, both with photographs, which show the different deformities met with in neurology, and with many excellent diagrams and several coloured plates. If only for the illustrations, the book is one which should form a very useful addition to the usual text-book.

**Medical Inspection of School Children in Bristol.** Bristol. 1909.—We turn with interest to the Second Annual Report of the School Medical Officer to the Bristol Education Committee, because it gives for the first time an account of a full year of medical inspection of school children in Bristol. The report has been drawn up by Dr. T. A. Green, who is to be congratulated upon having compressed into a small space the data that are of interest and significance, without making his pages dull. A proper use of the available figures is very necessary, and a perusal of this report shows that the medical inspection of school children is destined to be of national value, not merely by reason of its direct effect in the early detection of disease, but also in that it will in time provide a suitably large stock of data for various statistical inquiries. For example, we are given a number of interesting tables of average heights and weights at different ages, from which it appears that Bristol children are a little shorter and a good deal lighter than average British children of corresponding ages, and that the older children are of better physique if they have been brought up in one of the less densely populated areas of the city than those who have enjoyed less favourable conditions. A beginning has also been made with the question of the relation of a high infant mortality to the physique of the survivors : this is, of course, not to be settled by one year's observations, so that for conclusions we must wait till the inquiry which is opened by this report has covered a longer period. Turning to the nature of the various physical defects discovered by medical examination, we find that out of 15,161 children examined 271 had to be excluded from school either permanently or for varying periods on account of divers diseases. The principal items in this category were non-tuberculous pulmonary affections, pulmonary tuberculosis, scabies, impetigo, chorea, epilepsy, enlarged tonsils and adenoids. Defects of some kind were noted in 7,249 children, or nearly half of those examined. In 1,691 instances parents were advised of the need for medical treatment which did not involve absence from school. Of these, 698 were cases of enlarged tonsils and adenoids, or of adenoids only. These figures do not include certain defects and disorders. Vermin of the scalp, for example, were notified in 72 boys and 2,208 girls: in the extermination of these pests the medical inspectors received much valuable help from the two school nurses, whose whole time was devoted to visiting children in their homes, and helping to carry out such treatment as was needful. Of 8,379 children whose vision was tested, 856 were found defective in this respect; the local education authorities have made arrangements for the supply of cheap spectacles to scholars who need them. Definite mental defect was noted in 43 cases, actual imbecility in 5, and 426 were found to be "dull." One or more decayed teeth were noted in nearly two-thirds of 11,597 children examined for this purpose. In about half of these cases dental treatment was recommended. Infectious disease must have been responsible for very many absences from school: there were 729 cases of scarlet fever and diphtheria among school children in the year, and we are interested to see that no less than 2,050 cases of non-notifiable infectious disease (including 1,126 of measles) were notified by head teachers to the medical officer of health. It is a little disappointing to find that parents are not more alive to the advantages which their children enjoy from systematic inspection. Only 57 per cent. of the children examined were accompanied by parents, but it is interesting to note that the district in which parents attended best is the poorest in the city. It is definitely known that no action was taken by the parents in about one-third of the cases of general disease in children fit to attend school, in about half the cases of eye defect, and in nearly two-thirds of the cases of dental defect in which treatment was recommended. Such apathy is extraordinary and lamentable, but it is to be hoped that as the value of inspection is demonstrated by series of concrete instances the parents will awake to the fact that they are getting a great deal for nothing, and that they are wasting precious opportunities. In conclusion, attention must be drawn to the interesting notes at the end of the report by the medical officers to special schools, Dr. L. M. Griffiths and Dr. J. S. Griffiths. The whole report is full of interest, and it is clear that the school medical officers are working with an enthusiasm which cannot fail to tell favourably on the health of the community in the long run.

**The Medical Annual.** Twenty-eighth year. Bristol: John Wright & Sons, Ltd. 1910.—The new work of the year has been in this volume described by thirty-two contributors, three of whom are American. The editor asks, "Have we, in sea water, a plasma which will supply the protoplasmic cells with something the lack of which causes certain chronic diseases? How far will the 'hormones' carry us in the cure of some diseases when drugs have failed? Will the bacterial vaccines enable us to check the invasion of the bacilli? These are questions of the immediate future, and we have done our best to place existing knowledge before our readers." We notice that bacterial vaccines, sea water and internal secretions have not quite displaced drugs from the position they have previously occupied; there are still seventy-nine pages of therapeutic progress, but it is gratifying to learn that the output of new drugs is falling off, for otherwise the new drugs would exceed the number of patients on whom they have to be tried. The advances in surgical technique and accurate diagnosis occupy much space, and the subjects of typhoid fever, cancer and syphilis have received full attention. The plates showing the various urinary deposits are no better than those which were found in the text-books of fifty years ago, and we fail to see on what ground the editors accepted the suggestion of a correspondent who should have been sufficiently familiar with these deposits. An immense amount of useful information is bound up in the volume, which continues to be well up to the latest views of things, and must be really indispensable to the busy practitioner who can only read a limited amount of current medical literature. It should be on the study table for constant reference, and will be found a reliable and suggestive friend in emergencies. We are glad to see that stereoscopic plates are not out of date; a picture which is not stereoscopic is scarcely worthy of inspection.

**Transactions of the Thirty-third Annual Meeting of the American Dermatological Association, 1909.**—Though we have come to expect that matters of interest will be dealt with at the Annual Meeting of the American Dermatological Association, the *Transactions* for 1909 are unusually rich in matters of recent advance in dermatology. Much use has been made by various observers of the Wassermann reaction in syphilis, and of Noguchi's modification of this test. Though it may take long before implicit reliance can be laid upon tests of this nature, the compiling of facts by competent pathologists can only prove of service. Thus when Whitehouse draws attention to five out of seven cases of scleroderma which were positive to Wassermann's test, he may or may not be throwing light upon the pathology of the complaint, but he certainly awakens interest in a possible connection between some forms of this disease and syphilis. Psoriasis is dealt with in three interesting papers by Pollitzer, Schamberg and Breakey, while Fordyce treats of the pathology and histological appearances of the Lichen group of skin diseases. An interesting paper by White records his observation on the elastic tissue of the skin. The *Transactions* are concluded by the usual interesting summary of the dermatological returns from the various clinics, which comprise some 36,500 cases.

**Catalogue of the Pathological Museum of the University of Manchester.** By J. LORRAIN SMITH, M.A., M.Sc., M.D., F.R.S. Manchester: At the University Press.—This admirably arranged catalogue is a model of what a museum catalogue should be, and it would be a great advantage were every medical museum to issue a similar publication. The reviewer has found it very useful when searching for examples of rare lesions, and it ought to find a place on the bookshelf of every department of morbid anatomy. The Council of the University of Manchester have set a valuable precedent in making their collection of pathological specimens widely available, and it is to be hoped that other Councils may be equally generous. To Professor Lorrain Smith and his collaborators we offer our unstinted praise and thanks. The energy and time consumed in the preparation of the volume must have been enormous. Medical literature is much enhanced by such a sterling contribution as this.

**Male Diseases in General Practice.** By EDRED M. CORNER, M.A., M.C. Pp. xvi, 462. London: Oxford Medical Publications. 1910.—This is an excellent book, and deserves the attention of the student and general practitioner. It records many valuable observations and statistics obtained by the author and others from a vast number of cases. The chapters dealing with hydrocele and the imperfectly descended testicle are particularly good in the many practical points and original suggestions that are presented. Torsion of the testicle is dealt with in a more thorough and complete manner, as the result of modern observations, than is commonly found in many current text-books. Of particular interest is the work done by the author on the cremasteric reflex and its value as an indication of general and local conditions of disease. Without hesitation, one can recommend the book as a whole.

**Lectures on Surgical Nursing.** By E. STANMORE BISHOP, F.R.C.S. Pp. 143. Bristol: John Wright & Sons, Ltd. 1909. —This book accomplishes its object, the aim of which is to supply the surgical nurse with the reasons for the modern methods of treatment in surgical cases, in order to enable her to co-operate intelligently with the surgeon; instructing her in the many details of nursing that are essential to success, and setting forth in an orderly and readable manner her duties before, during and after operation.

**How to Cut the Drug Bill.** A. HERBERT HART, M.D. London: Bale, Sons and Danielsson. 1910.—To the dispensing practitioner this booklet should appeal strongly, as the author claims to have reduced his drug bill by 50 per cent. in acting on the methods laid down in the first edition. He proposes to bring out an annual edition of his work.

